# *Lactiplantibacillus plantarum* CCNH185 Attenuates *Citrobacter rodentium*-Induced Colitis by Reshaping Gut Microbiota Structure and Modulating Innate Immunity

**DOI:** 10.3390/foods15101815

**Published:** 2026-05-20

**Authors:** Yizhi Jing, Xiaoyue Bai, Yuanzhi Yin, Xinfeng Liu, Junzhu Li, Zhichao Chen, Zhengyuan Zhai, Yanling Hao

**Affiliations:** 1 Key Laboratory of Precision Nutrition and Food Quality, Department of Nutrition and Health, China Agricultural University, Beijing 100193, China; j15502414985@163.com (Y.J.); 15933668553@163.com (X.L.); 2 College of Food Science and Nutritional Engineering, China Agricultural University, Beijing 100083, China; baixiaoyue21@163.com (X.B.); yyz_17@163.com (Y.Y.); ljz19961020@163.com (J.L.); 18380368719@163.com (Z.C.); zhaizy@cau.edu.cn (Z.Z.)

**Keywords:** probiotic, infectious colitis, mucus barrier, inflammation

## Abstract

Infectious enteritis caused by bacterial pathogens are a significant global health concern, with high incidence and mortalities worldwide. The objective of this research was to explore the benefits of *Lactiplantibacillus plantarum* CCNH185 against *Citrobacter rodentium*-induced colitis in mice. Female C57BL/6J mice (*n* = 8 per group) were orally administered *L. plantarum* CCNH185 at a dose of 2 × 10^9^ CFU daily for 24 days, followed by a single oral challenge with *C. rodentium* (2 × 10^9^ CFU) on day 21. *L. plantarum* CCNH185 significantly alleviated disease symptoms including body weight loss, colon shortening and histopathological damage (*p* < 0.05). Treatment with *L. plantarum* CCNH185 also reduced pro-inflammatory cytokine levels, such as *IL-1β* and *IL-6* (*p* < 0.05), while increasing anti-inflammatory *IL-10* expression (*p* < 0.05) in the colon. Histological and immunofluorescence demonstrated that *L. plantarum* CCNH185 improved the intestinal barrier integrity by increasing goblet cell numbers, upregulating MUC2 expression, reducing crypt hyperplasia, and suppressing epithelial cell apoptosis. Furthermore, transcriptomic analysis revealed that *L. plantarum* CCNH185 suppressed excessive immune cell infiltration and inflammatory responses in the colon during *C. rodentium* infection. Flow cytometry analysis further confirmed that *L. plantarum* CCNH185 suppressed hyperactivation of innate immune cells including macrophages, dendritic cells, neutrophils to alleviate inflammation. Furthermore, *L. plantarum* CCNH185 reshaped the gut microbiota by increasing the abundance of beneficial genera such as *Lactobacillus*, *Dubosiella*, and *Romboutsia*. Correlation analysis linked these microbial shifts with improved inflammatory and apoptotic markers. These findings highlight *L. plantarum* CCNH185 may serve as a promising preventive probiotic candidate for ameliorating infectious colitis possibly through strengthening the gut mucus barrier, modulating immune responses, and altering gut microbiota composition.

## 1. Introduction

Gastrointestinal infections caused by bacterial pathogens remain a major worldwide health challenge, with high incidence and mortalities burden globally [1]. Enteric pathogen infections can lead to a wide range of diseases, such as infectious colitis, post-infectious irritable bowel syndrome, hemolytic uremic syndrome, and sepsis [2,3]. During infection, attaching and effacing (A/E) pathogens, such as enteropathogenic *Escherichia coli* (EPEC) and enterohemorrhagic *E. coli* (EHEC) use the type III secretion system (T3SS) to deliver effector proteins into host epithelial cells, reprogramming inflammatory signaling, innate defense, and cell death pathways [4]. This promotes breakdown of the gut barrier together with an exaggerated activation of the mucosal immune system, thereby facilitating pathogen invasion. Although antibiotics remain the primary treatment for enteric pathogen infection, their use is often associated with significant limitations, including disturbance of the intestinal microbial community, increased susceptibility to secondary infections and the incidence of hemolytic uremic syndrome, as well as the emergence of antibiotic resistance [5]. Therefore, probiotics have been recognized as a promising non-antibiotic approach for the prevention and treatment of enteric pathogen infection.

Accumulating evidence suggests that probiotics can alleviate enteric infection through various mechanisms, such as pathogen competitive exclusion, intestinal barrier function improvement and immune system regulation [6]. For instance, *Weissella cibaria* LAB_Weis_Camel_L4 mitigated *E*. *coli*–induced enteritis by competitively excluding pathogens and modulating gut microbiota composition [7]. *L. plantarum* CCFM8661 could enhance the intestinal barrier function by increasing the expression of tight junction proteins (Claudin-1, Occludin, and ZO-1) and alleviate diarrhea symptoms in mice infected with *E. coli* [8]. *Lacticaseibacillus paracasei* L4 increased goblet cells, along with the expression of ZO-1, Occludin, and Claudin-1, alleviate the *E. coli* O8 infection in mice [9]. *Bifidobacterium bifidum* FL228.1 increased numbers of IgA^+^ cells, reduced ETEC load, alleviated damage to intestinal tissue and attenuated systemic inflammation in an *E. coli*-infected weanling mouse model [10]. *L. plantarum* NWAFU-BIO-BS29 alleviated *E. coli*-induced colitis by mitigating the inflammatory reaction and re-modulating the intestinal microbiota in BALB/c mice [11]. Despite these promising findings, probiotic actions remain highly species-specific or even strain-specific, highlighting the importance of mechanistic investigations to identify and validate strains with the capacity to alleviate enteric infection.

In this study, *L. plantarum* CCNH185, isolated from koumiss—a traditional fermented dairy product—was selected based on its previously demonstrated antimicrobial activity against enteric pathogens, including *Escherichia coli*, *Salmonella*, and *Helicobacter pylori*, as well as its anti-inflammatory effects in murine colitis model [12]. Despite these promising findings, the specific mechanisms by which *L. plantarum* CCNH185 protects against *C. rodentium*-induced infectious colitis remain largely unexplored. *Citrobacter rodentium*, a natural murine pathogen that forms A/E lesions similar to those induced by human pathogens, is commonly employed as a standard model for investigating host–pathogen interactions and mucosal immune responses in the gut [13]. Therefore, we hypothesized that preventive administration of *L. plantarum* CCNH185 ameliorates *C. rodentium*-induced colitis through modulation of the gut microbiota and regulation of mucosal immune responses. The results demonstrated that *L. plantarum* CCNH185 was found to ameliorate intestinal inflammation, enhance the intestinal mucus barrier, and modulate innate immunity and composition of the gut microbiota. These findings highlight the potential of *L. plantarum* CCNH185 to modulate mucosal immunity and provide a basis for further investigation of its potential role in managing infectious enteritis.

## 2. Materials and Methods

### 2.1. Preparation of Bacterial Culture Suspension

The *L. plantarum* CCNH185 was provided by COFCO Corporation (Beijing, China). *L. plantarum* CCNH185 was sub-cultured three times in de Man, Rogosa, and Sharp medium (Beijing Aobox Biotechnology Co., Ltd., Beijing, China) using a 2% (*v*/*v*) inoculum and incubated at 37 °C for 24 h. *Citrobacter rodentium* ATCC 51459 was purchased from Ningbo Mingzhou Biotechnology Co., Ltd. (Ningbo, China) and grown in Luria-Bertani broth at 37 °C for 14 h at 150 rpm. The bacterial pellet was collected by centrifugation at 3000× *g* for 10 min at 4 °C. The cell pellets were washed once with sterile phosphate-buffered saline (PBS, pH 7.4) and subsequently resuspended in PBS to obtain a final concentration of 1 × 10^10^ CFU/mL before administration.

### 2.2. Animal Experimental Design

**Figure 1:**
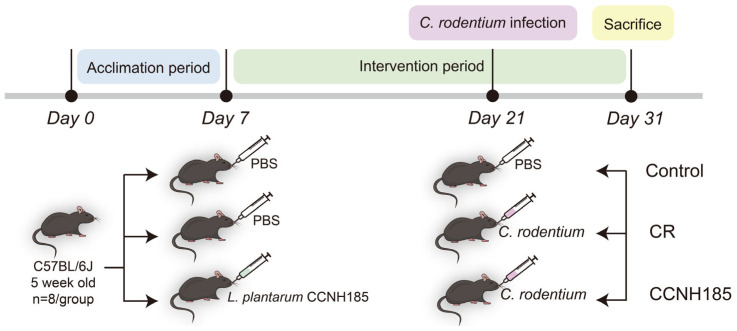
Experimental design.

Female C57BL/6J mice (five weeks old; 18–20 g) were sourced from Vital River Laboratory Animal Technology Co., Ltd. (Beijing, China). All animals were housed under specific-pathogen-free (SPF) conditions (22 ± 2 °C, 50–70% humidity, 12 h light/dark cycle) and fed standard chow (Sipeifu Biotechnology Co., Ltd., Beijing, China) with water ad libitum. Following a 7-day acclimation period, mice were randomly assigned to three groups (*n* = 8 per group, a total of 24 mice) including control, CR (*C. rodentium*) and CCNH185 (*L. plantarum* CCNH185) groups (Figure 1). From day 8 through day 31, mice in the control and CR groups were orally gavaged with 0.2 mL of sterile Phosphate-Buffered Saline (PBS) once daily, whereas those in the CCNH185 group were administered 2 × 10^9^ CFU of *L. plantarum* CCNH185 per day. On day 21, mice in CR and CCNH185 groups were orally administered with 2 × 10^9^ CFU *C. rodentium*, whereas mice in the control group received an equal volume of sterile PBS. On day 30, fresh fecal samples were collected from all mice for subsequent 16S rRNA sequencing analysis. On day 31, all mice were euthanized, and blood was collected via retro-orbital puncture.

### 2.3. Histological Analysis and Immunofluorescence

Colonic tissues fixed in 4% paraformaldehyde for 24 h were paraffin-embedded, sectioned at 3–4 μm, and then stained with hematoxylin and eosin (H&E) and alcian blue periodic acid-schiff (AB-PAS) according to standard protocols. Histological images were acquired using a light microscope. Histopathological injury was scored in a blinded manner according to an established index (Table S1) that grades the severity of inflammation and the extent of involvement. Colonic tissue sections for immunofluorescence were dewaxed, rehydrated, and then treated with citric acid antigen retrieval buffer to retrieve antigens. Sections were then incubated with primary antibodies against Ki-67 (1:250, 16667, Abcam, Cambridge, UK), MUC2 (1:3000, 27675-1-AP, Proteintech, Wuhan, China) and CD4 (1:50, SC-19641, Santa Cruz, Dallas, TX, USA). Following washing, sections were incubated with species-specific Alexa Fluor-conjugated secondary antibodies at room temperature for 1 h in the dark. After nuclear counterstaining with DAPI, fluorescence images were captured under a fluorescence microscope, and the fluorescence intensity was semi-quantitatively analyzed using ImageJ V1.51 software (Bethesda, MD, USA).

### 2.4. Assessment of Apoptosis by TUNEL Assay

Apoptotic cells in colonic tissues were detected using a terminal deoxynucleotidyl transferase-mediated dUTP nick-end labeling (TUNEL) assay kit (Beyotime Biotechnology, Beijing, China) according to the manufacturer’s instructions. Briefly, colon sections were permeabilized with 0.1% Triton X-100 (2 min, room temperature), then incubated with TUNEL reaction mixture (1 h, 37 °C) and finally counterstained with DAPI (1 min, 25 °C). Fluorescence images were captured using a fluorescence microscope under identical exposure settings. Apoptotic cell counts were obtained from five random microscopic fields per mouse, with intervals of about 100 μm between fields.

### 2.5. Quantitative Reverse Transcription PCR

Colon tissues weighing 25 mg were cleaned with cold PBS and stored at −80 °C to enable RNA isolation. Total RNA was isolated using AG RNAex Pro reagent (Accurate Biotechnology Hunan Co., Ltd., Changsha, China). The RNA quantity and quality were then accurately assessed by the NanoDrop ONEc Spectrophotometer (Thermo Fisher Scientific, Waltham, MA, USA). After generating cDNA from 1 µg of total RNA with the Evo M-MLV RT Mix Kit (Accurate Biotechnology Hunan Co., Ltd., Changsha, China), qRT-PCR was performed using the SYBR Green Premix Pro Taq Hs qPCR kit on a QuantStudio™ 5 Real-Time PCR System (Thermo Fisher Scientific, Wilmington, DE, USA). All the primer used in this study are listed in Table S2. Relative quantification of the target gene was calculated using *β-actin* as the reference gene according to the 2^−∆∆CT^ method.

### 2.6. Transcriptomic Analysis

Total RNA was isolated from colon tissues using commercial extraction kits (D4015, Omega Bio-Tek, USA). Sequencing libraries were prepared using the Illumina TruSeq RNA sample preparation kits (Illumina, San Diego, CA, USA), following the manufacturer’s guidelines. Briefly, the RNA concentration and quality were evaluated using a Nanodrop 2000 spectrophotometer (Thermo, Waltham, MA, USA) and agarose gel electrophoresis. mRNA was selectively enriched using oligo dT-conjugated beads, followed by the fragmentation and isolation of fragments with approximately 300 base pairs in length through magnetic beads. The cDNA synthesis, amplification, and sequencing were performed according to a standardized protocol. After cluster generation, the libraries were subjected to sequencing on the Illumina NovaSeq 6000 system (Illumina, San Diego, CA, USA). Differentially expressed genes and their correlation network analysis were conducted on the Majorbio cloud platform (https://www.majorbio.com/).

### 2.7. Isolation of Colonic Lamina Propria Cells

Colonic lamina propria cells were isolated for subsequent flow cytometric analysis as previously described with minor modifications [14]. Briefly, freshly collected colons were cut into 3 cm segments, opened longitudinally, and thoroughly washed with ice-cold PBS until all luminal contents were removed. To remove the epithelial layer, the tissue fragments were incubated in pre-warmed 0.5 mM EDTA at 37 °C for 30 min with continuous shaking at 220 rpm. After incubation, residual epithelial cells were removed by vigorous shaking in ice-cold PBS. The supernatant containing epithelial cells was discarded. Then, the epithelial-free fragments were transferred to a digestion solution consisting of 0.08 U/mL Dispase II (Solarbio, D6430, Beijing, China) and 300 U/mL Collagenase II (Gibco, 17101-015, Waltham, MA, USA) dissolved in serum-free DMEM/F-12 medium. The samples were then incubated at 37 °C for 30 min with horizontal rotation at 180 rpm. Following enzymatic digestion, the cell suspension was further dissociated by vigorous agitation in ice-cold PBS. After removal of undigested debris by filtering the cell suspension through a 40-μm nylon mesh, cell pellets were obtained via centrifugation (500× *g*, 5 min, 4 °C). The resulting cell pellets were re-suspended in ice-cold cell staining buffer and kept on ice until further analysis.

### 2.8. Flow Cytometry

To minimize nonspecific antibody binding, single-cell suspensions were first incubated with flow cytometry receptor blocking reagent (BioLegend, 101302, San Diego, CA, USA) on ice for 10 min. Cells were stained with the fluorochrome-conjugated antibody panel listed in Table S3 at 4 °C for 20 min in the dark. After washing and centrifugation (500× *g*, 5 min, 4 °C) of the stained samples, the cell pellets were resuspended in 500 µL of ice-cold cell staining buffer. To discriminate between live and dead cells, Hoechst 33342 was added, and the cells were incubated on ice for 15 min. Data acquisition was performed immediately using a CytoFLEX flow cytometer (Beckman Coulter, Brea, CA, USA). The data were analyzed using FlowJo software (v10.8.1), with proper gating strategies employed for the exclusion of debris, doublets, and dead cells (Figure S1).

### 2.9. Gut Microbiota Analysis

Fecal samples were subjected to 16S rRNA microbial analysis at Majorbio Bio-Pharm Technology Co. Ltd. (Shanghai, China). Briefly, fecal DNA was extracted using the E.Z.N.A.^®^ Soil DNA Kit (Omega Bio-tek, Inc., Norcross, GA, USA), and the bacterial 16S rRNA gene was amplified with bar-coded universal primers 27F-1492R. Amplicons were quantified on a Quantus™ fluorometer and performed with paired-end (2 × 300 bp) sequencing on an Illumina MiSeq PE300 platform. Demultiplexed reads were uploaded to Majorbio Cloud (https://cloud.majorbio.com) for taxonomic assignment and differential-abundance profiling analysis against host phenotypes.

### 2.10. Statistical Analysis

Statistical analysis was performed using GraphPad Prism 9.0. Data are presented as the mean ± SD. The normality of the data was assessed using the Shapiro–Wilk test, and all data showed normal distribution (*p* > 0.05 for each group). Levene’s test was also performed to verify equality of variances. Inter-group differences were evaluated by one-way analysis of variance (ANOVA), followed by Dunnett’s post hoc test, with the Control group serving as the reference. For gut microbiota analysis, alpha diversity indices were compared using the Kruskal-Wallis test followed by Dunn’s post-hoc test with Benjamini-Hochberg false discovery rate (FDR) correction. Beta diversity was assessed by principal coordinate analysis (PCoA) based on Bray-Curtis dissimilarity. Differentially abundant taxa were identified by LEfSe (linear discriminant analysis effect size), with an LDA threshold > 3.0. Correlations between bacterial abundances and host parameters were calculated using Spearman’s rank correlation coefficient, and the Benjamini-Hochberg FDR correction was applied. *p* < 0.05 was considered statistically significant.

## 3. Results

### 3.1. L. plantarum CCNH185 Ameliorated C. rodentium-Induced Colitis

**Figure 2:**
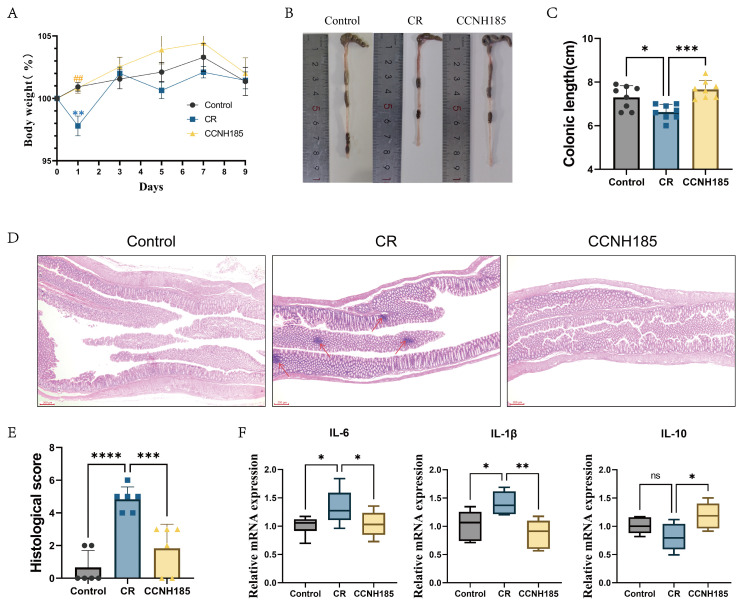
(**A**) Body weight changes. (**B**) Representative colon images. (**C**) Colonic length. (**D**) Typical H&E-stained colon sections from each group (scale bar = 200 µm). Arrows indicate: epithelial damage and inflammatory cell infiltration. (**E**) Histological score. (**F**) The mRNA levels of *IL-6*, *IL-1β*, *IL-10* in colon tissues. (##: *p* < 0.01 vs. CR group; ns indicates not significant, * *p* < 0.05, ** *p* < 0.01, *** *p* < 0.001, and **** *p* < 0.0001).

To assess the protective effects of *L. plantarum* CCNH185 against *C. rodentium*-induced colitis in C57BL/6J, *L. plantarum* CCNH185 was administered for 14 days before *C. rodentium* exposure. Compared with the control group, body weight was significantly decreased in the CR group on day 1 post-infection (Figure 2A). However, *L. plantarum* CCNH185 significantly attenuated this weight loss. The colon length was significantly shorter in the CR group (6.6 cm) than in the control group (Figure 2B,C). Notably, *L. plantarum* CCNH185 intervention significantly increased the colon length to 7.6 cm. Histopathological analysis further showed inflammatory cell infiltration and epithelial damage in the CR group, accompanied by significantly increased histological scores (Figure 2D,E). However, *L. plantarum* CCNH185 treatment significantly reduced these damage scores. Furthermore, the mRNA expression level of *IL-6* and *IL-1β* was significantly decreased, and the *IL-10* mRNA expression level was significantly increased in the CCNH185 group (Figure 2F). These results indicate that *L. plantarum* CCNH185 treatment alleviated *C. rodentium*-induced colitis in mice.

### 3.2. L. plantarum CCNH185 Restores Intestinal Mucus Barrier Integrity

**Figure 3:**
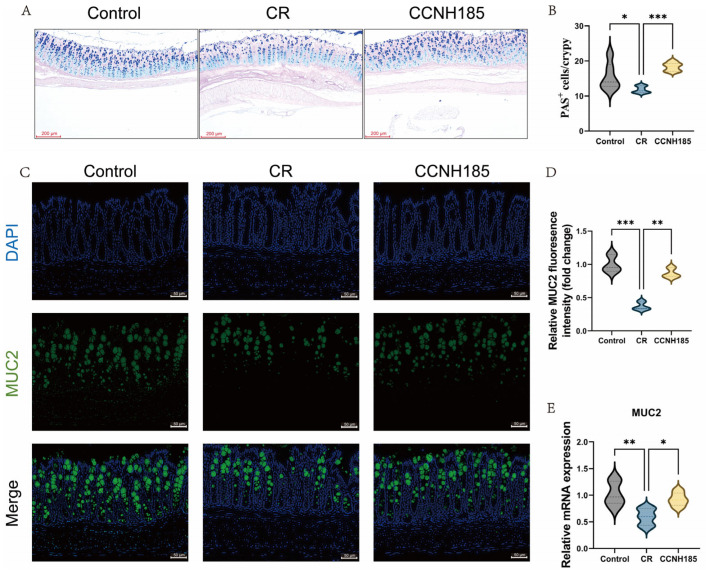
Analysis of colonic tissues by histology and immunofluorescence. (**A**) Representative AB-PAS staining of colon sections in each group (scale bar = 200 µm). (**B**) Quantification of goblet cells per crypt. (**C**) Representative MUC2 immunofluorescence (green), with nuclei counterstained with DAPI (blue) (scale bar = 50 μm). (**D**) Quantification of the MUC2 fluorescence intensity. (**E**) The mRNA levels of *MUC2* in colon tissues. (* *p* < 0.05, ** *p* < 0.01, and *** *p* < 0.001).

To investigate the effect of *L. plantarum* CCNH185 on the intestinal mucus barrier, AB-PAS staining was performed on colon tissues in this study (Figure 3A). A significant decrease in goblet cells was observed in the CR group (11.8 per crypt) relative to the control group (Figure 3B). *L. plantarum* CCNH185 intervention could increase the goblet cells to 18.3 cells per crypt. Furthermore, immunofluorescence analysis confirmed that *L. plantarum* CCNH185 significantly increased MUC2 expression compared to the CR group (Figure 3C,D). Meanwhile, *L. plantarum* CCNH185 treatment also increased the *MUC2* mRNA expression level (Figure 3E). These results indicate that *L. plantarum* CCNH185 protected against *C. rodentium*–induced damage to the colonic mucus barrier by promoting goblet cell abundance and MUC2 production.

### 3.3. L. plantarum CCNH185 Attenuates Aberrant Proliferation and Apoptosis of Colonic Epithelial Cells

**Figure 4:**
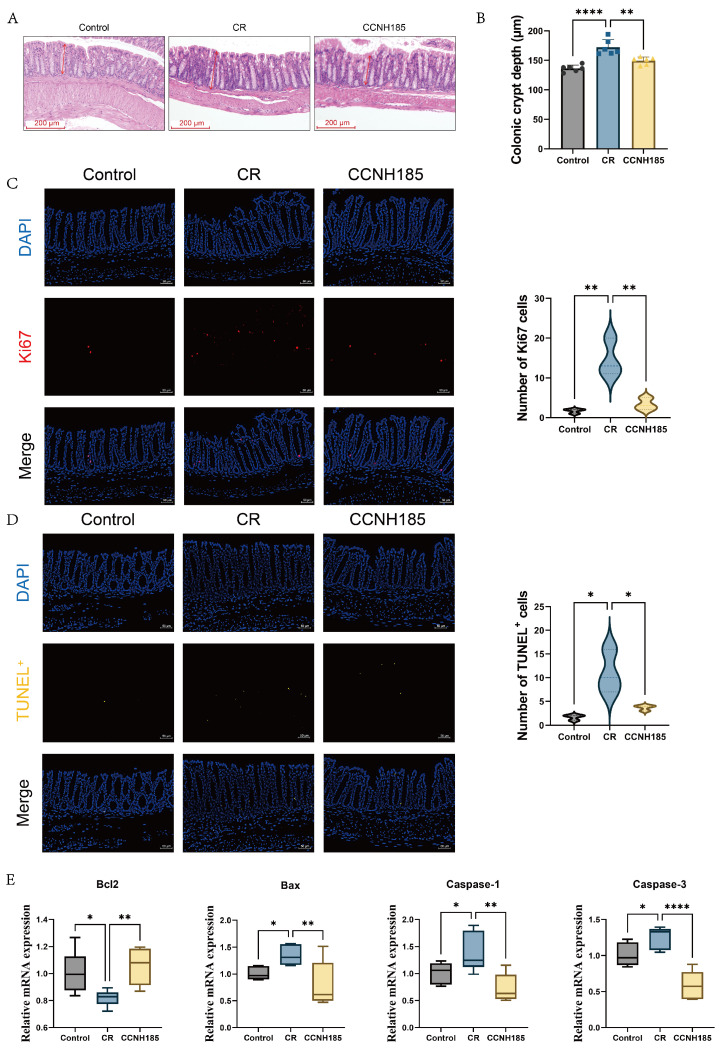
*L. plantarum* CCNH185 mitigates crypt hyperplasia in the colon. (**A**) H&E staining of representative colonic sections from each experimental group (scale bar = 200 µm). Arrows indicate crypt depth. (**B**) Quantification of colonic crypt depth. (**C**) Representative immunofluorescence staining of Ki67^+^ (red) cells, with nuclei counterstained with DAPI (blue) (scale bar = 50 μm). Quantification of the Ki67+ cells. (**D**) Representative immunofluorescence staining of TUNEL^+^ (yellow) cells, with nuclei counterstained with DAPI (blue) (scale bar = 50 μm). Quantification of the TUNEL+ cells. (**E**) The mRNA levels of *Bcl2*, *Bax*, *Caspase-1*, and *Caspase-3* in colon tissues. (* *p* < 0.05, ** *p* < 0.01, and **** *p* < 0.0001).

The protective effect of *L. plantarum* CCNH185 on epithelial homeostasis was evaluated by assessing colonic crypt architecture, cell proliferation, and apoptosis. The colonic crypt depth significantly increased to 172 μm in the CR group, indicative of epithelial hyperplasia following *C. rodentium* infection (Figure 4A,B). In contrast, *L. plantarum* CCNH185 treatment significantly reduced crypt depth to 149 μm. Notably, immunofluorescence analysis revealed an 8.8-fold significant increase in Ki67^+^ cells in the CR group relative to the control group (Figure 4C). *L. plantarum* CCNH185 treatment significantly reduced Ki67^+^ cell abundance compared with the CR group. Furthermore, TUNEL staining demonstrated an increase in apoptosis cells in the colonic tissue of the CR group (6.6-fold vs. control) (Figure 4D). *L. plantarum* CCNH185 treatment significantly reduced TUNEL^+^ cell numbers in the colon compared with the CR group. Meanwhile, the mRNA expression of the anti-apoptotic gene *Bcl2* was markedly down-regulated in the CR group, and pro-apoptotic genes *Bax*, *Caspase-1* and *Caspase-3* were upregulaed by 1.38 and 1.25-fold, respectively (Figure 4E). *L. plantarum* CCNH185 intervention significantly restored the expression levels of these corresponding genes. These findings indicate that *L. plantarum* CCNH185 restores epithelial homeostasis by modulating proliferation and apoptosis in response to *C. rodentium* infection, thereby maintaining intestinal barrier integrity.

### 3.4. L. plantarum CCNH185 Enhanced Epithelial Repair While Suppressing Excessive Immune Response

**Figure 5:**
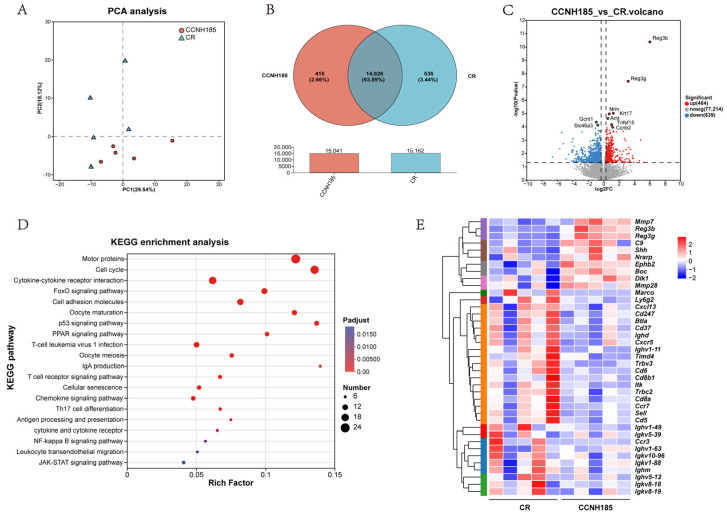
Transcriptional analysis showing the effects of CCNH185 on *C. rodentium*-induced colitis. (**A**) Principal component analysis (PCA) plot of CCNH185 and CR group; (**B**) Venn diagram showing the overlap of expressed genes between CCNH185 and CR group; (**C**) Volcano plot illustrates the genes that were differentially expressed in the CCNH185 group compared to the CR group (fold change > 1.25, P-adjust < 0.05); (**D**) KEGG pathway enrichment analysis of DEGs, highlighting significantly affected biological pathways; (**E**) Heatmap of expression profiles for DEGs associated with immune response, anti-infection and intestinal repair.

To investigate the molecular mechanisms by which *L. plantarum* CCNH185 alleviates *C. rodentium*-induced colitis, we performed transcriptome profiling of colonic tissues from CR model and CCNH185-treated mice. Principal component analysis (PCA) showed a distinct separation between the CR and CCNH185 groups (Figure 5A), indicating substantial CCNH185-treated transcriptional alterations. Venn diagram analysis revealed that 14,626 genes were commonly expressed between the CCNH185 and CR groups, representing 93.89% of the total expressed genes (Figure 5B). A total of 1103 genes showed significant alterations in transcript levels between the CCNH185 and CR groups, including 464 that were upregulated and 639 that were downregulated (|Fold Change| > 1.25, P-adjust < 0.05, Figure 5C). KEGG enrichment analysis of CCNH185-regulated differential expression genes (DEGs) revealed significant representation of immunity-related pathways, including cytokine-cytokine receptor interaction, cell adhesion molecules, antibody production and T cell receptor signaling (Figure 5D).

Among these DEGs, antimicrobial genes were strongly upregulated in the CCNH185 group compared with the CR group, including *Reg3b* (65.31-fold), *Reg3g* (9.26-fold), *C9* (65.31-fold), and *Mmp7* (65.31-fold), indicating enhanced intestinal antibacterial defense (Table S4). In addition, several genes involved in epithelial repair and maintenance of intestinal homeostasis were upregulated, including *Shh* (2.13-fold), *Boc* (1.33-fold), *Nrarp* (1.34-fold) and *Dlk1* (2.42-fold), which participate in Hedgehog- and Notch-related signaling pathways regulating progenitor cell differentiation and epithelial regeneration. Conversely, multiple genes involved in immune cell activation and infiltration were significantly downregulated. These included B-cell and immunoglobulin-related genes (*Ighd*, *Ighm*, *Ighv1-49*, etc., −4.63 to −51.80 fold), chemokines and chemokine receptors (*Cxcr5*, *Ccl28*, *Ccr3*, etc., −1.50 to −16.65 fold), macrophage receptor *Marco* (−37.19 fold), as well as T-cell receptor signaling genes (*Trac*, *Trbv3*, *Itk*, etc., −2.31 to −11.40 fold). Furthermore, several markers related to lymphocyte activation and adhesion, including *Cd247*, *Cd8a*, *Cd5*, *Cd6*, *Cd37*, *Btla*, *Sell*, and *Ly6g2*, were also significantly reduced after CCNH185 intervention (Figure 5E and Table S4). Together, these transcriptomic changes indicate that CCNH185 enhanced antimicrobial defense and epithelial repair while suppressing excessive immune cell infiltration and inflammatory responses in the colon during *C. rodentium* infection.

### 3.5. L. plantarum CCNH185 Suppressed Hyperactivation of Innate Immune Cells

**Figure 6:**
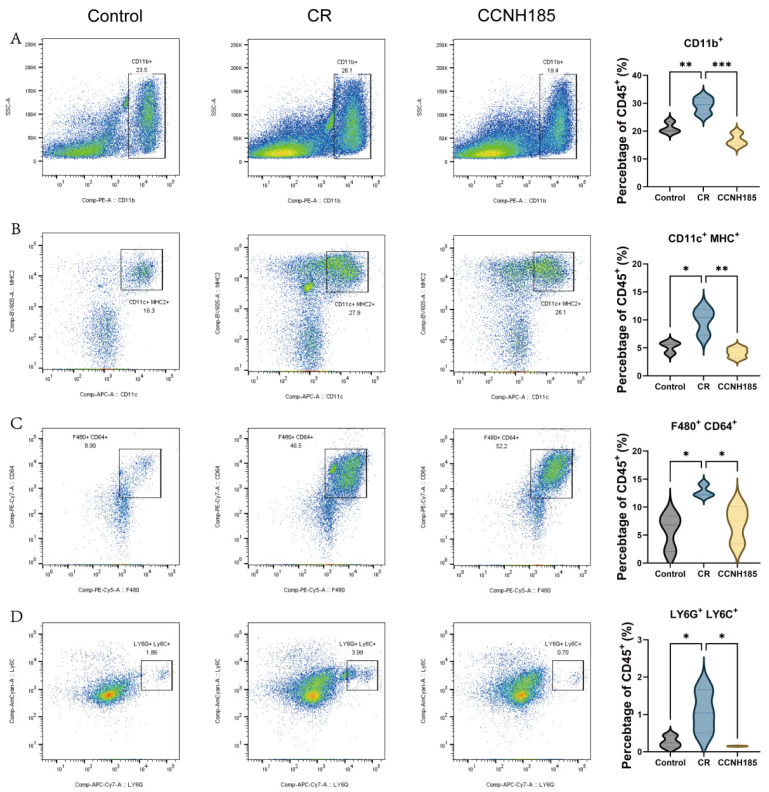
Statistical analysis of colon lamina propria inflammatory cells. (**A**) Myeloid cells (CD11b^+^). (**B**) Dendritic cells (CD11c^+^MHC^+^). (**C**) Macrophages (F480^+^CD64^+^). (**D**) Neutrophils (LY6G^+^LY6C^+^). (* *p* < 0.05, ** *p* < 0.01, and *** *p* < 0.001).

To validate the transcriptomic findings, immune cells from the colonic lamina propria were isolated and analyzed by flow cytometry. Post-dissociation cell viability was greater than 80%, and at least 7 × 10^5^ total cells were acquired for analysis, including a minimum of 4 × 10^4^ CD45^+^ leukocytes for downstream analysis. The results showed that myeloid cells (CD11b^+^), dendritic cells (CD11c^+^MHC^+^), macrophages (F4/80^+^CD64^+^) and neutrophils (LY6G^+^LY6C^+^) were significantly increased in the CR group (Figure 6A–D). This indicated that *C. rodentium* induced excessive activation of innate immune cells in the colonic lamina propria. In contrast, *L. plantarum* CCNH185 treatment significantly reduced the accumulation of myeloid cells, dendritic cells, macrophages and neutrophils. These results demonstrated that *L. plantarum* CCNH185 alleviates *C. rodentium*-induced colitis, at least in part, by suppressing the hyperactivation of innate immune cells in the colonic lamina propria.

### 3.6. L. plantarum CCNH185 Ameliorated Colitis in Mice by Modulating Gut Microbiota Composition

**Figure 7:**
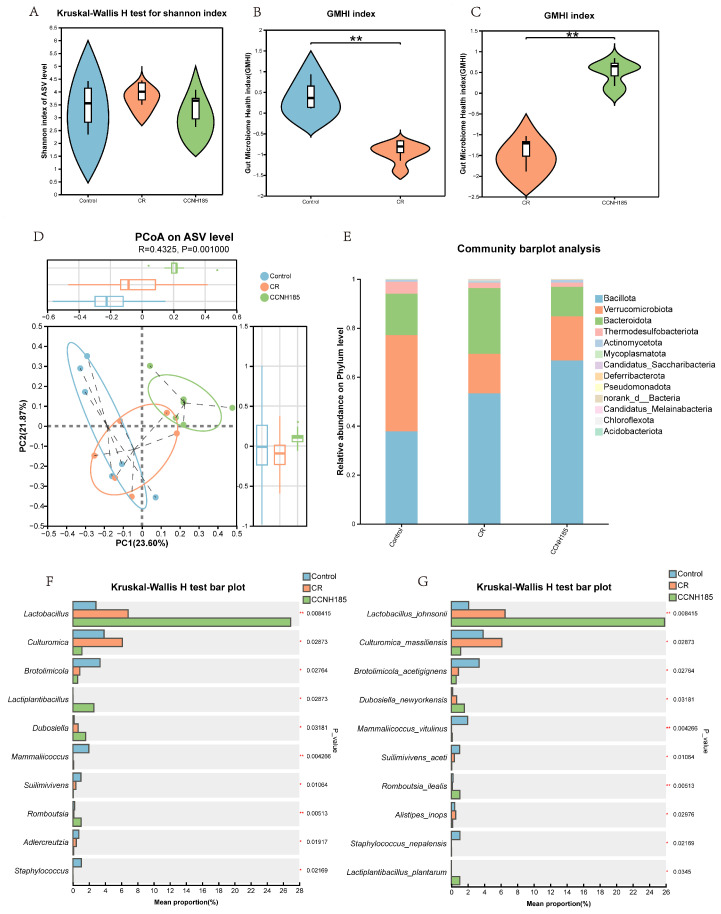
*L. plantarum* CCNH185 restores the intestinal microbial community in *C. rodentium*-infected mice. (**A**) Shannon index of Alpha diversity. (**B**,**C**) Gut microbial health index at the ASV level. (**D**) β-Diversity based on principal co-ordinates analysis. (**E**) Relative abundance of bacteria at the phylum level. (**F**) Genus-level analysis of gut microbial composition comparing the CR group with the CCNH185 group. (**G**) Species-level analysis of gut microbial composition comparing the CR group with the CCNH185 group. (* *p* < 0.05 and ** *p* < 0.01).

To investigate the effect of *L. plantarum* CCNH185 on gut microbiota composition, 16S rRNA sequencing was performed in this study. The α-diversity based on Shannon index showed no significant differences among groups (Figure 7A). However, the gut microbiota health index was significantly reduced in the CR group (Figure 7B). In contrast, this index was significantly higher in the CCNH185 group than in the CR group (Figure 7C). β-diversity analysis showed that *L. plantarum* CCNH185 treatment significantly changed microbiota composition (Figure 7D). At the phylum level, Bacteroidota abundance increased in the CR group but decreased in the CCNH185 group (Figure 7E). In addition, Verrucomicrobiota abundance was reduced in the CR group and restored upon CCNH185 treatment. At genus-level, the abundance of *Culturomica* was significantly increased in the CR group (Figure 7F). *L. plantarum* CCNH185 treatment significantly reduced *Culturomica* abundance. In addition, CCNH185 increased the relative abundances of beneficial genera, including *Lactobacillus*, *Lactiplantibacillus*, *Dubosiella*, and *Romboutsia* (Figure 7F). Specifically, *L. plantarum* CCNH185 significantly increased the abundance of *Lactobacillus_johnsonii*, *Dubosiella_newyorkensis*, *Romboutsia_ilealis*, and *Lactiplantibacillus_plantarum* (Figure 7G).

**Figure 8:**
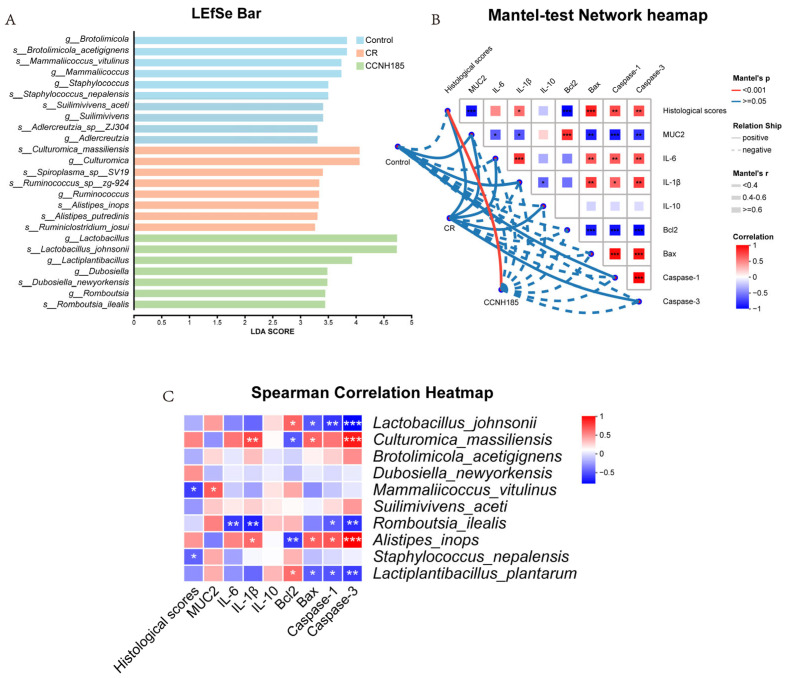
(**A**) LDA scores of microbial taxa enriched as identified by LEfSe analysis (LDA > 3). (**B**) Mantel Test network heatmap analysis. Box color corresponded to the strength of correlation coefficients. (**C**) Heatmap visualizing the correlations between species-level bacterial taxa and multiple biochemical parameters. (* *p* < 0.05, ** *p* < 0.01, and *** *p* < 0.001).

To further characterize microbiota alterations associated with *L. plantarum* CCNH185 intervention, differentially abundant taxa among groups were identified using linear discriminant analysis effect size (LEfSe). LDA analysis (LDA > 3) showed that *Culturomica_massiliensis*, *Acetivibrio_aldrichii*, *Spiroplasma* sp. *SV19*, *Bacteroides_uniformis*, *Ruminococcus* sp. *zg-924*, and *Alistipes_inops* were significantly enriched in the CR group (Figure 8A). In contrast, *Lactobacillus johnsonii*, *Dubosiella newyorkensis*, and *Romboutsia ilealis* were identified as key functional species in the CCNH185 group. Mantel test network heatmap showed significant correlations between histological scores and multiple inflammatory and apoptotic markers (Figure 8B). Specifically, histological damage was negatively correlated with *MUC2* and *Bcl2* expression and positively correlated with pro-inflammatory genes *IL-6* and *IL-1β* and pro-apoptotic genes *Bax*, *Caspase-1*, and *Caspase-3*. Spearman correlation analysis further indicated that the expression of pro-apoptotic genes was negatively correlated with the abundances of *Lactobacillus johnsonii*, *Romboutsia ilealis*, and *Lactiplantibacillus plantarum*, but positively correlated with the abundances of *Culturomica massiliensis* and *Alistipes inops* (Figure 8C). These results demonstrate that *L. plantarum* CCNH185 ameliorated *C. rodentium*-induced colitis by modulating the gut microbiota composition.

## 4. Discussion

Colitis induced by pathogenic infection is typically characterized by pronounced mucosal inflammation, crypt hyperplasia, and increased epithelial apoptosis. In particular, *C. rodentium* infection is characterized by crypt hyperplasia, elevated Ki67^+^ epithelial proliferation, and increased epithelial apoptosis and inflammation [15]. A growing body of evidence demonstrates that probiotic interventions can effectively ameliorate *C. rodentium*-induced colitis. For instance, treatment with the yeast probiotic *S. boulardii* alleviates *C. rodentium* induced colitis by decreasing numbers of mucosal adherent *C. rodentium*, ameliorating crypt hyperplasia, and maintaining intestinal epithelial barrier function [16]. Pretreatment with a mixture of *L. helveticus* R0052 and *L. rhamnosus* R0011 attenuated mucosal inflammation, epithelial hyperplasia, and colonic epithelial apoptosis in *C. rodentium*–induced colitis [17]. Consistent with these findings, *L. plantarum* CCNH185 treatment restored the Ki67^+^ cells level to that of the control group. *L. plantarum* CCNH185 treatment markedly reduced epithelial apoptosis, as indicated by decreased TUNEL^+^ cells and modulation of apoptosis-related genes (*Bcl2*, *Bax, Caspase-1*, and *Caspase-3*). These results indicate that CCNH185 effectively re-establishes epithelial homeostasis, thereby contributing to the overall amelioration of colitis.

Epithelial homeostasis is associated with intestinal barrier integrity, particularly the mucus layer, which protects against enteric pathogen invasion. Pathogenic infection often disrupts the mucus layer or reduces mucin secretion, thereby facilitating pathogen invasion and triggering inflammation. Generally, increased goblet cell numbers and enhanced mucin production contribute to strengthening the mucus barrier, which limits pathogen adherence to the epithelium and promotes pathogen clearance. Probiotics have been reported to augment mucus barrier function by increasing goblet cell differentiation and mucin production [18,19]. For example, *L. plantarum* NWAFU-BIO-BS29 could upregulate *Muc2* expression and strengthen mucus layer integrity, thereby alleviating *E. coli*–induced colitis [11]. In this study, *L. plantarum* CCNH185 reinforces mucus barrier by restoring goblet cell numbers and increasing *MUC2* expression. The restoration of mucus barrier integrity likely represents an early and critical step in limiting infection progression and promoting intestinal recovery.

*C. rodentium* infection also leads to excessive activation of mucosal immunity. This is characterized by increased recruitment and activation of innate immune cells in the lamina propria, including myeloid cells, dendritic cells, macrophages, and neutrophils. The overactivation of innate immune cells results in excessive secretion of inflammatory mediators, which causes tissue damage and compromises the gut barrier. Previous studies have shown that probiotic interventions can effectively suppress excessive mucosal immune activation. For example, Lactobacillus johnsonii attenuates *C. rodentium*-induced colitis by reducing immune cell infiltration and proinflammatory cytokine secretion [14]. *L. acidophilus* NCFM could enhance dendritic cell activity, which in turn strengthens mucosal immune responses and reduces the severity of *C. rodentium*-induced colitis [20]. Our transcriptomic analysis revealed that *L. plantarum* CCNH185 significantly downregulated the expression of chemokines and their receptors, such as *Cxcr5* and *Ccl28*, which are involved in immune cell recruitment. Additionally, *L. plantarum* CCNH185 reduced the expression of *Marco*, a macrophage receptor associated with NF-κB mediated pro-inflammatory responses, suggesting suppression of pro-inflammatory phagocytic activity. Furthermore, genes related to B cell function, immunoglobulin production, and T cell receptor signaling were also downregulated, indicating attenuation of adaptive immune activation. These findings were further supported by flow cytometry results, which showed that *L. plantarum* CCNH185 significantly reduced the proportions of myeloid cells, dendritic cells, macrophages, and neutrophils in the colonic lamina propria. Together, these results demonstrate that *L. plantarum* CCNH185 effectively suppresses excessive innate immune activation and restores immune homeostasis.

Given the close interplay between mucosal immunity and gut microbial communities, the gut microbiota is essential for preserving intestinal barrier integrity and orchestrating immune homeostasis [21,22]. *C. rodentium* infection disrupts the gut microbiota composition and reduces the abundance of beneficial bacteria [23]. In the present study, *L. plantarum* CCNH185 improved the gut microbiota health index and altered microbial community structure, as evidenced by β-diversity analysis. Notably, *L. plantarum* CCNH185 intervention increased the abundance of *Lactobacillus* and *Lactiplantibacillus*. These bacterial genera known to suppress pathogenic bacteria and enhance mucus barrier integrity [24]. *L. plantarum* contributes to the amelioration of *C. rodentium*-associated pathology and Th1 cell dysregulation [25]. *L. johnsonii* shows systemic effects on both innate and adaptive immune cell populations in human investigations [26]. In addition, *L. johnsonii*-derived extracellular vesicles enhance polarization of M2 macrophages and maintenance of the intestinal barrier [27]. Moreover, the abundance of *D. newyorkensis* and *R*. *ilealis* was significantly increased in the CCNH185 group. *D. newyorkensis* is a bacterium that can prevent DSS-induced colitis and reduce the expression of inflammatory factors [28]. *D. newyorkensis* has been identified as playing an important role in restoring the Treg/Th17 balance and alleviating mucosal barrier damage via propionate and L-lysine generation [29]. And *R. ilealis* enhances the immune system by activating the intestinal TLR2/NF-κB signaling pathway [30]. In this study, *R. ilealis* also showed significant negative correlations with *IL-6*, *IL-1β*, *Caspase-1*, and *Caspase-3*, supporting its anti-inflammatory and anti-apoptotic roles. Collectively, these findings point to a role for *L. plantarum* CCNH185-mediated microbiota restructuring in diminishing inflammatory responses and epithelial apoptosis.

In addition, several limitations of this study should be acknowledged. A CCNH185-only group without *C. rodentium* infection was not included. However, our primary aim was to evaluate the protective effect of the probiotic under infectious conditions, and the absence of this group does not undermine the observed efficacy of CCNH185 in alleviating colitis. Furthermore, we did not quantify *C. rodentium* load, measure microbial metabolites, or evaluate tight junction proteins. Nonetheless, the marked improvement in histopathological and inflammatory parameters indicates a clear protective outcome. The microbiota data are correlational, and causal links require functional validation. This study represents a preventive intervention rather than a therapeutic one. Future studies should perform comparative metabolomics, secretome analysis, and functional assays using other *L. plantarum* strains to establish strain-specific novelty.

## 5. Conclusions

In this study, *L. plantarum* CCNH185 effectively alleviated *C. rodentium*-induced colitis in mice. Treatment with *L. plantarum* CCNH185 protected against the disruption of the colonic mucus barrier by promoting goblet cell abundance and enhancing MUC2 production. Additionally, *L. plantarum* CCNH185 restored epithelial homeostasis by regulating cell proliferation and apoptosis in response to *C. rodentium* infection, thereby maintaining intestinal barrier integrity. Transcriptomic analysis revealed that *L. plantarum* CCNH185 enhanced antimicrobial defense and epithelial repair, while suppressing excessive immune cell infiltration and inflammatory responses in the colon. Furthermore, *L. plantarum* CCNH185 modulated the gut microbiota composition, which might contribute to reduced inflammation and epithelial apoptosis. Overall, these findings highlight the potential of *L. plantarum* CCNH185 as a promising preventive probiotic for managing infectious colitis by strengthening the mucus barrier, modulating immune responses, and reshaping the gut microbiota.

## Data Availability

The original contributions presented in this study are included in the article/Supplementary Material. Further inquiries can be directed to the corresponding author.

## Supplementary Materials

The following supporting information can be downloaded at: https://www.mdpi.com/article/10.3390/foods15101815/s1, Figure S1: Gating strategy for identification of innate immune cells; Figure S2: The absolute numbers of CD45^+^ cells in each group; Table S1: Histological scores of colon damage; Table S2: QPCR primers used in this work; Table S3: Antibody used in this work; Table S4: Gene differential expression in colon tissue between CCNH185 and CR group.
