# Supplementary material for: Lactiplantibacillus plantarum CCNH185 Attenuates Citrobacter rodentium-Induced Colitis by Reshaping Gut Microbiota Structure and Modulating Innate Immunity
Source: Foods. 2026 May 20;15(10):1815. doi: 10.3390/foods15101815 (PMC13206718; doi:10.3390/foods15101815)
Supplement: Supplementary file 1 [file foods-15-01815-s001.zip › foods-4306976-supplementary.pdf]

Supplement materials

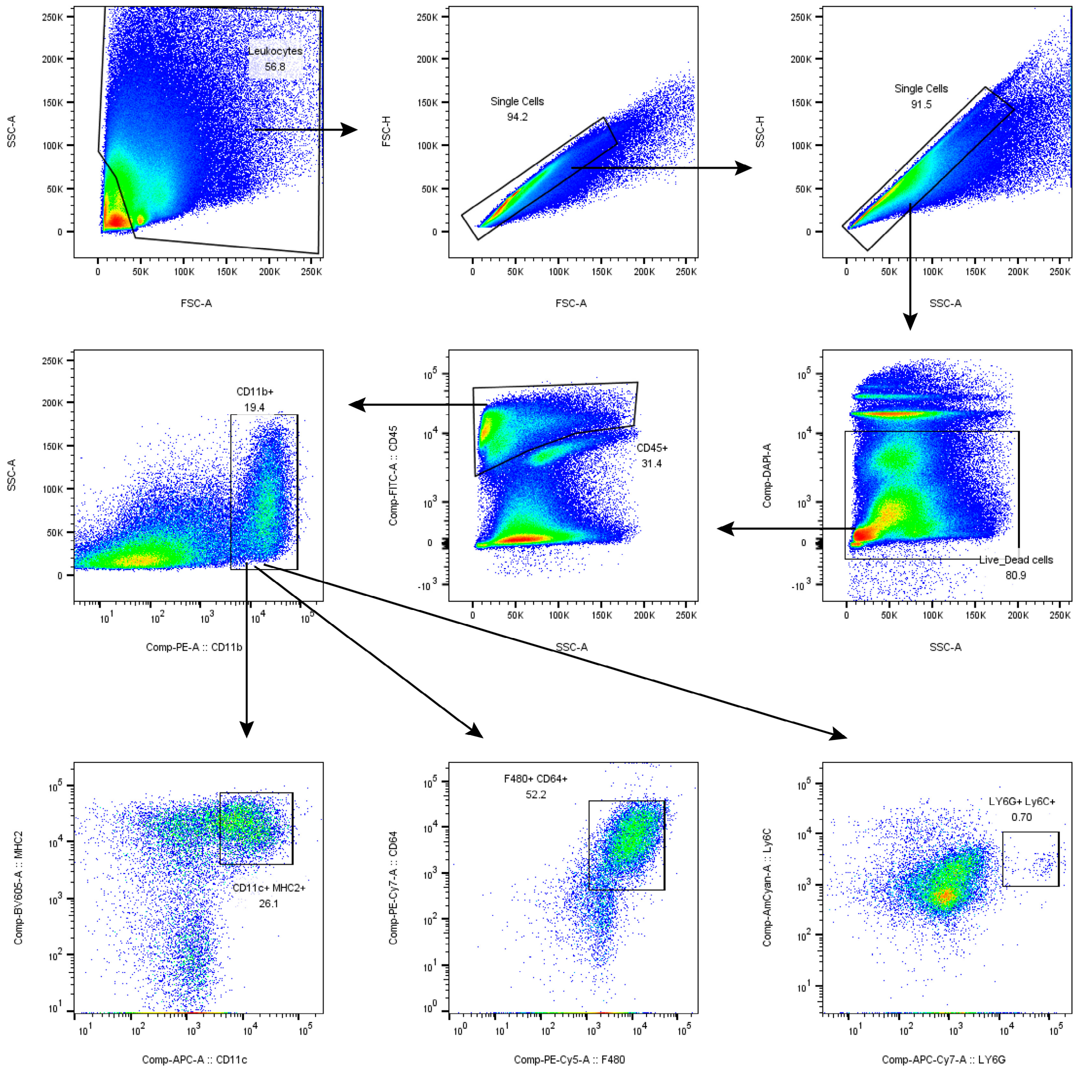

Figure S1. Gating strategy for identification of innate immune cells.

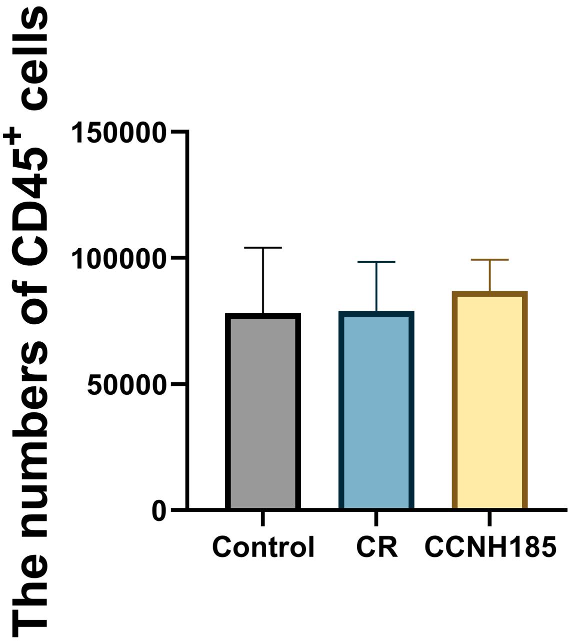

Figure S2. The absolute numbers of CD45⁺ cells in each group.

Table S1. Histological scores of colon damage[a].

Score	Inflammation	Extent
0	None	None
1	Slight	Mucosa
2	Moderate	Mucosa and Submucosa
3	Severe	Transmural colonic tissues

[a]Total histological score = inflammation severity score + extent score (range 0–6).

Table S2. QPCR primers used in this work.

**Gene**	**Forward primer(5′-3′)**	**Reverse primer (5′-3′)**
β-actin	GTGACGTTGACATCCGTAAAGA	GCCGGACTCATCGTACTCC
MUC2	ATGCCCACCTCCTCAAAGAC	GTAGTTTCCGTTGGAACAGTGAA
IL-6	CCAAGAGGTGAGTGCTTCCC	CTGTTGTTCAGACTCTCTCCCT
IL-1β	GCAACTGTTCCTGAACTCAACT	ATCTTTTGGGGTCCGTCAACT
IL-10	GCTCTTACTGACTGGCATGAG	CGCAGCTCTAGGAGCATGTG
Bcl2	GTCGCTACCGTCGTGACTTC	CAGACATGCACCTACCCAGC
Bax	TGAAGACAGGGGCCTTTTTG	AATTCGCCGGAGACACTCG
Caspase-1	ACTCGGGTGGAAGAAGACAG	CTCCTCAGCAAATCGGAACTG
Caspase-3	TGGTGATGAAGGGGTCATTTATG	TTCGGCTTTCCAGTCAGACTC

Table S3. Antibody used in this work.

**Antibody**	**Brand**	**Product Number**
$CD11b^+$	BioLegend	101207
$CD11C^+$	BioLegend	117309
$MHC2^+$	BioLegend	107639
$F4/80^+$	BioLegend	123111
$CD64^+$	BioLegend	139313
$LY6G^+$	BioLegend	127623
$LY6C^+$	BioLegend	128033

Table S4. Gene differential expression in colon tissue between CCNH185 and CR group[a].

Category	Gene name	Description	Fold Change (CCNH185 vs CR)
Anti-infection	*Reg3b*	regenerating islet-derived 3 beta	65.31
	Reg3g	regenerating islet-derived 3 gamma	9.26
	C9	complement component 9	4.73
	Mmp7	matrix metallopeptidase 7	2.74
Epithelial regeneration	*Shh*	sonic hedgehog	2.13
	Boc	BOC cell adhesion associated, oncogene regulated	1.33
	Mmp28	epilysin	1.60
	Nrarp	Notch-regulated ankyrin repeat protein	1.34
	Dlk1	delta like non-canonical Notch ligand 1	2.42
	Ephb2	Eph receptor B2	1.28
Immune response	*Ighd*	immunoglobulin heavy constant delta	-4.63
	Ighm	immunoglobulin heavy constant mu	-4.64
	Igkv8-19	immunoglobulin kappa variable 8-19	-5.67
	Igkv8-18	immunoglobulin kappa variable 8-18	-6.60
	Ighv5-12	immunoglobulin heavy variable 5-12	-7.15
	Igkv1-88	immunoglobulin kappa chain variable 1-88	-7.57
	Ighv1-11	immunoglobulin heavy variable V1-11	-11.32
	Ighv1-63	immunoglobulin heavy variable V1-63	-11.65
	Igkv10-96	immunoglobulin kappa variable 10-96	-14.22
	Igkv5-39	immunoglobulin kappa variable 5-39	-16.78
	Ighv1-49	immunoglobulin heavy variable 1-49	-51.80
	Cxcl13	C-X-C motif chemokine ligand 13	-2.56
	Cxcr5	C-X-C motif chemokine	-2.86

	receptor 5	
Ccl28	C-C motif chemokine ligand 28	-1.50
Ccr3	C-C motif chemokine receptor 3	-16.65
Marco	macrophage receptor with collagenous structure	-37.19
Trac	T cell receptor alpha constant	-2.97
Trbc2	T cell receptor beta, constant 2	-2.52
Trbv3	T cell receptor beta, variable 3	-11.40
Itk	IL2 inducible T cell kinase	-2.31
Timd4	T cell immunoglobulin and mucin domain containing 4	-3.28
Ly6g2	lymphocyte antigen 6 family member G2	-1.74
Btla	B and T lymphocyte associated	-2.59
Sell	selectin, lymphocyte	-6.52
Cd247	CD247 antigen	-2.85
Cd8a	CD8 subunit alpha	-3.17
Cd8b1	CD8 subunit beta 1	-5.33
Cd5	CD5 antigen	-3.32
Cd6	CD6 antigen	-2.79
Cd96	CD96 antigen	-2.37
Cd37	CD37 antigen	-2.08

[a] Representative differentially expressed genes
